# Resilience characteristics and prior life stress determine anticipatory response to acute social stress in children aged 7–11 years

**DOI:** 10.1111/bjhp.12353

**Published:** 2019-01-13

**Authors:** Tara J. Cheetham‐Blake, Julie M. Turner‐Cobb, Hannah E. Family, James E. Turner

**Affiliations:** ^1^ Department of Psychology University of Bath UK; ^2^ Department of Pharmacy and Pharmacology University of Bath UK; ^3^ Department of Health University of Bath UK

**Keywords:** child, coping, cortisol, resilience, social support, stress testing

## Abstract

**Objectives:**

To assess the interplay of prior life stress and characteristics of resilience in determining how children cope with potentially stressful situations, using a two‐phase study that triangulates parent–child dyadic interview data with subsequent experience of an acute laboratory stressor in 7–11‐year‐olds.

**Methods:**

Participants (*n* = 34) were designated as being in one of four groups based on high/low levels of prior stress experience and high/low resilience ratings assessed during at‐home interviews and from questionnaires measuring recent life events, hassles, and trait coping. During a subsequent laboratory stress protocol, salivary cortisol and heart rate were monitored, and a verbal subjective report was provided.

**Results:**

Salivary cortisol showed a significant increase in anticipation of the stress test, heart rate increased during the test, and children self‐reported the task as stressful. Males displayed higher levels of cortisol than females in the anticipatory period. We observed no increase in salivary cortisol in response to the stress testing phase. Using the stress/resilience categorization, children with a higher level of resilience were differentiated by cortisol level in anticipation of the acute stress experiment based on their level of prior life stress. Highly resilient children with greater experience of prior life stress showed a lower anticipatory cortisol response than highly resilient children with less experience of prior life stress.

**Conclusions:**

This study highlights the relevance of contextual factors, such as prior stress experience and resilience, in physiological response to the anticipation of acute stress and has implications for understanding how children cope with stressful experiences.

**Statement of contribution**

***What is already known on this subject?***

An adaptation to the stress testing paradigm, the Bath Experimental Stress Test for Children (BEST‐C) was found to reliably induce a salivary cortisol response in young children, suggesting that peer matching the audience was an effective modification to laboratory social stress testing. Recent work focusing on early life adversity has seen the emergence of prior stress experience and resilience as key factors in the examination of acute stress responses. However, much of the research regarding the impact of childhood stress is ambiguous; some research suggests that if children have experienced prior stressful life events this will enact a positive effect on stress responses and lead to resilience, and other research suggested that it will have a compounding negative effect.

***What does the study add?***

- Findings provide support for the capacity of the BEST‐C to induce an anticipation stress response in children.
- Contextual factors e.g., prior stress experience and resilience are key for understanding stress responses.
- Resilient children with more experience of stress show lower cortisol than those with less stress experience.

## Background

Recent work focusing on early life adversity has seen the emergence of prior stress experience and resilience as key factors in the examination of acute stress responses. For example, past stressful experiences can have a compounding effect when combined with acute stressors (Marin, Chen, Munch, & Miller, 2009; Michaud, Matheson, Kelly, & Anisman, 2008). However, much of the research regarding the impact of childhood stress is ambiguous; some research suggests that if children have experienced prior stressful life events, this will enact a positive effect on stress responses and lead to resilience. For example, experiencing stress associated with the natural transition to school has been found to have a positive impact on endocrine activation and acute health outcomes (Turner‐Cobb, Rixon, & Jessop, 2011). Whereas other research suggests that stress can have negative consequences for cognitive functioning, emotional, and physical development (Ehlert, 2013). One key difference between these studies is the type of stressor encountered, with the former examining school transition and the latter trauma and adverse living conditions, suggesting that more extreme stressors are associated with negative outcomes and milder stressors with positive future stress responses. The response to acute social stressors encountered in childhood is crucial since repeated or accumulated stress experience influences the development of hypothalamic–pituitary–adrenal (HPA) axis functioning and the setting of the HPA axis for subsequent health into adulthood (Essex *et al*., 2011).

Resilience is defined as the ability to overcome stress or adversity, an ability which can develop over time through the interactions between a person and their environment (Egeland, Carlson, & Sroufe, 1993; Masten, 2014). Resilience characteristics such as coping ability can be moderators of responses to the Trier Social Stress Test (TSST; Abelson *et al*., 2014) and as such stress–resilience characteristics deserve attention in stress testing paradigms. As resilience concerns the interaction between a person and their environment, personality, and temperament factors (such as social support and competence) are important facets to examine (Bai & Repetti, 2015; Egeland *et al*., 1993; Lavoie, Pereira, & Talwar, 2014; Smith & Prior, 1995). Coping ability and resources are integral to the development of resilience. Numerous conceptualizations of coping exist within the literature, for example the transactional model of coping (Lazarus & Folkman, 1984), control models (Rothbaum, Weisz, & Snyder, 1982), and hierarchical models (Krohne, 1996). More recent research with young children has used factor analysis to develop a three‐factor model of coping (Turner‐Cobb & Steptoe, 1998) This data‐led model included problem‐focussed and emotion‐focussed coping, as in the transactional model of coping, but also highlighted avoidant coping as a third factor.

Social stress tests such as the TSST are a reliable method of inducing stress in adults (Kirschbaum, Pirke, & Hellhammer, 1993). Such testing usually involves public speaking and mental arithmetic in front of an audience, which triggers feelings of threat to the social self, known as social evaluative threat (Dickerson, Gruenewald, & Kemeny, 2009; Dickerson & Kemeny, 2004; Gunnar, Talge, & Herrera, 2009). This leads to activation of the HPA axis and the release of cortisol, most frequently assessed via saliva sampling.

Modifications to the TSST have made it suitable for use with children, for example the TSST‐C was adapted for children aged 9–14 years old and involved completing an unfinished story (Buske‐Kirschbaum *et al*., 1997), and the TSST‐M was modified for children aged 9–12 years by reducing the duration of the tasks and asking children to imagine they had started a new school and had to introduce themselves to a new class (Yim, Quas, Cahill, & Hayakawa, 2010). These versions of the TSST have been successful in eliciting a salivary cortisol response in children; however, one review reports that 12 out of 17 studies showed an increase in salivary cortisol (Gunnar *et al*., 2009), suggesting that approximately one‐third of stress tests do not induce a stress response. One possible explanation for these mixed findings is the standardized use of an adult audience to induce social evaluative threat. In adult stress testing, participants complete the tasks in front of an audience of their peers. Similarly, in child stress testing, the participants have, until recently, undertaken the stress task in front of an adult audience. This creates a power dynamic in child stress testing that may not be present in adult stress testing and could explain the discrepancies in responses to these tasks. The Bath Experimental Stress Test for Children (BEST‐C) was developed to increase comparability across child and adult stress testing (Cheetham & Turner‐Cobb, 2016). Using a child audience, it makes the stress test more ecologically valid as peer interaction and evaluation is a prominent feature of children's life experience. The BEST‐C was found to reliably induce a salivary cortisol response in young children, suggesting that peer matching the audience was an effective modification to laboratory social stress testing (Cheetham & Turner‐Cobb, 2016).

The aim of the current study was to assess the interplay of prior life stress and characteristics of resilience in determining how children cope with potentially stressful situations, using a two‐phase study that triangulates parent–child dyadic interview data with subsequent experience of an acute laboratory stressor in 7–11‐year‐olds. Further developing our understanding of which factors are protective and which are detrimental has implications for children's stress management and cortisol responses across the life course. In the first phase of the study, children were categorized into one of four groups based on the dimensions of high/low levels of prior stress experience and high/low resilience ratings assessed during at‐home interviews and questionnaires assessing stressful life events, hassles, and coping strategies. In the second phase, children completed a laboratory social stress protocol, the BEST‐C (Cheetham & Turner‐Cobb, 2016), with salivary cortisol and heart rate monitored throughout, followed by a verbal subjective report. We hypothesized that salivary cortisol and heart rate would increase for all children in anticipation to and during the BEST‐C and that children with a combination of greater experience of prior stress and a higher level of resilience would show the smallest cortisol increase in response to the task.

## Methods

### Participants

Children aged 7–11 years old were recruited for a two‐phase study using an opt‐in recruitment method via local advertisements. Recruitment through an email to a local sports club was the most successful recruitment strategy, attracting almost half the participants in this study. The other half of the participants were recruited through school newsletters, newspaper adverts, emails to university staff, and the university website. Parent–child dyads who participated in at‐home interviews and questionnaires in the first phase of this study were invited to take part in the second phase using laboratory stress testing. The sample reported in this paper refers to the participants who participated in both the interviews and the stress testing protocol.

Thirty‐four children (19 males and 15 females) took part in the present investigation. Demographic details of the study sample are reported in Table 1. The sample size of 34 in the current study exceeds the G*Power recommendation of a sample size of 32 for an analysis using MANOVA with a medium effect size of 0.3, an alpha of 0.05 and power of 0.8 (Yim *et al*., 2010). The study was granted ethical approval from the University's Psychology research ethics committee.

**Table 1:** Totals (*N*), percentages (%), means, and standard deviations (*SD*) for the socio‐demographic details of the sample (*n* = 33)

	*N* (%)	Mean (*SD*)
Sex (*n*)		
Males	19 (58)	
Females	14 (42)	
Mean age (years)		8.91 (1.42)
Ethnicity (*n*)		
White British	27 (82)	
White European	3 (9)	
White British/Other	3 (9)	
Parent four‐factor SES score^a^		53.47 (9.02)

Note

a Possible socio‐economic status (SES) scores on this scale range from 8 to 66. Lower numbers correspond to lower SES, higher numbers to higher SES.

### Measures

#### Questionnaires

Socio‐demographic data (child age, sex, ethnicity, and parental socio‐economic status; SES) were collected alongside validated questionnaires to assess children's experiences of stressful life events, daily hassles, and coping strategies. The Social Readjustment Rating Scale (SRRS; Holmes & Rahe, 1967) was used to assess whether children had experienced any of the listed 31 stressful events in the past year (retest reliability of *r* = .71, Horowitz, Schaefer, Hiroto, Wilner, and Levin (1977)). This scale is widely used in the literature and was judged to be a valid measure of life events in a review assessing its use over 30 years (Scully, Tosi, & Banning, 2000). The Children's Hassles Scale (Kanner, Feldman, Weinberger, & Ford, 1987) was used to measure minor stressful events that had occurred in the past month (retest reliability of *r* = .79 for frequency of hassles, *r* = .48 for intensity, Kanner, Coyne, Schaefer, and Lazarus (1981)). This scale has been found to have high levels of predictive validity, with more frequent hassles associated with more emotional distress (Blount *et al*., 2008). The Kidcope questionnaire (Spirito, Stark, & Williams, 1988) measured which of 15 coping strategies children had used to deal with a past stressor (frequency of coping strategies) and which strategies they had found to be the most helpful (efficacy of coping strategies); retest reliability ranging from *r* = .56 to .75, Spirito *et al*. (1988). This scale was shown to be valid across a number of studies with children (Spirito, Stark, Gil, & Tyc, 1995; Spirito, Stark, & Tyc, 1989).

#### Phase 1 Interviews: At‐home parent–child dyadic interviews

Participants' responses to the questionnaires detailed above were used to guide semi‐structured interviews with each participant and one of their parents (parent–child dyad) about the topics of major and minor stressors, coping strategies, and resilience. The researcher examined the SRRS questionnaire and discussed with the participant the stressors they had listed to get a better understanding of their experiences of stress. This dyadic interview format allowed both child and parent to contribute to the discussion of stress. Analysis of this interview data is not reported here except for the description of how the questionnaire and interview data were used to categorize participants into groups based on high/low levels of prior stress experience and high/low resilience ratings.

#### Phase 2 Experimental social stress paradigm: The BEST‐C

The BEST‐C (Cheetham & Turner‐Cobb, 2016) is a laboratory social stress test based on the original TSST (Kirschbaum *et al*., 1993) and the modified version for children (TSST‐M; Yim *et al*., 2010). The BEST‐C involves a 10‐min task comprised of 6 min of public speaking (talking about themselves in the context of having started a new school) and 4 min of multiple subtraction (counting down from 825 in multiples of three) in front of an audience who respond negatively (e.g., looking at their mobile phone, yawning) to induce social evaluative threat. To make it more appropriate for young children, the BEST‐C uses a child panel rather than an adult panel which is projected as a ‘live video link’ rather than the panel being physically present in the room. The live video panel in the BEST‐C is pre‐recorded, ensuring that each participant receives identical responses from the panel.

#### Salivary cortisol sampling

Saliva samples were collected into 15‐ml centrifuge tubes using the passive drool technique and a 3‐min timed sample on five occasions. Samples were collected between 14:00 and 16:00 to account for the diurnal cortisol rhythm. The first sample (baseline) was provided by participants at home 24 hr before visiting the laboratory for stress testing. Participants and their parents were given detailed instructions to ensure that rigorous home baseline criteria were followed for the collection of this sample. Collecting a baseline sample on a control day, rather than on the same day as the stressor, is a technique recommended to reduce the impact of novelty and provide a better understanding of how an acute stressor impacts children's normal cortisol patterns (Lovallo, Farag, & Vincent, 2010; Wolfram, Bellingrath, Feuerhahn, & Kudielka, 2013).

The second sample (anticipation sample) was collected on the day of the stress test, after participants had arrived and had time to acclimatize to the laboratory, but before starting the 10‐min stress test. The third sample (stressor sample) was collected 10 min after the end of the BEST‐C to assess the peak cortisol response (Dieleman, van der Ende, Verhulst, & Huizink, 2010). The fourth and fifth samples (recovery samples) were collected 20 and 35 min after the end of the BEST‐C to capture the return of cortisol to baseline levels (Buske‐Kirschbaum *et al*., 1997).

The baseline sample was stored overnight at 4°C and brought to the laboratory by the participant. The remaining samples were stored at 4°C after collection and frozen at −20°C within 2 hr along with the baseline sample. Once the study data collection was complete, all samples were thawed, centrifuged at 2,500 *g* for 10 min to remove particulate matter, and the saliva was aliquoted into micro‐centrifuge tubes prior to assay. Salivary cortisol was measured in duplicate using commercially available enzyme immunoassay kits according to manufacturer instructions (Salimetrics salivary cortisol ELISA kit 1‐3002).

#### Heart rate monitoring

Recent research highlights the importance of measuring heart rate to better understand the stress‐induced cortisol response, especially during an anticipation period (Pulopulos, Vanderhasselt, & De Raedt, 2018). Heart rate was measured (beats per minute; bpm) using a finger oximeter (FOs2pro, Meditech). The oximeter was affixed to the participant's index finger on their non‐dominant hand for 30 min, spanning a baseline period (10 min prior to the BEST‐C), stressor period (10‐min task) and the beginning of the recovery period (10 min after the task).

### Procedure

Written consent was given by the accompanying parent and verbal assent was given by each child. In the first phase of the study, children, with the assistance of their parents, completed the demographic, life events, daily hassles, and coping questionnaires. The questionnaire responses were then used to guide semi‐structured dyadic interviews with children and their parents. Participants completed the questionnaires and interview with the researcher on the same day. After the interviews, participants were given the option to take part in the second phase of the study. If they agreed, a date in the next 2 weeks was booked in for them to come to the laboratory.

In the second phase of the study, parents collected the baseline saliva sample from their child approximately 24 hr prior to attending the laboratory to undertake the BEST‐C. Children were accompanied to the laboratory by one of their parents. After a period of acclimatization to the laboratory, participants provided a saliva sample (anticipation sample) and had the heart rate monitor attached to their finger. Children were given 10 min to prepare for the public speaking aspect of the BEST‐C. During the BEST‐C, participants were asked to stand in front of the audience (the researcher was present in the room, whereas the child panel members were projected as a life‐sized image on a screen).

Following the BEST‐C, participants were escorted to a debrief room to relax and re‐join their parent. Ten minutes post‐test, children provided a third saliva sample and the heart rate monitor was removed. The fourth and fifth saliva samples were collected 20 and 35 min post‐test. Children were debriefed about the deception used in the study (i.e., the audience of children was pre‐recorded) and discussed their experience of the stress test with the researcher. The post‐test verbal report is a feature of the BEST‐C protocol, and as such acted as an elaborated manipulation check to assess children's self‐reported stress and coping in response to the BEST‐C.

#### Quantitatively coding the interviews into four prior stress–resilience groups

The questionnaires and interviews conducted in phase one of this study were used to categorize participants into groups based on their prior experience of stress and resilience characteristics. Four groups were created as follows: high resilience/high prior stress (eight participants), high resilience/low prior stress (10 participants), low resilience/high prior stress (nine participants), and low resilience/low prior stress (seven participants). These groupings are based on the categorization procedure of research investigating resilience and vulnerability to stress in adolescents (D'Imperio, Dubrow, & Ippolita, 2000).

To establish children's level of prior stress experience (high or low), the number and severity of the stressors mentioned in the interviews were counted and compared to the summed scores from the major life events and daily stressors questionnaires. Participants were split into groups based on the median score. This mixed methods triangulation demonstrated high congruence between the quantitative and qualitative data. Thematic analysis of the qualitative data was carried out but is not reported here (manuscript in preparation).

Children were split into high and low resilience groups based on the resilience factors discussed in the interviews. These factors were tabulated for each participant and a decision as to a participant's level of resilience (high or low) was made based on the number and importance of the resilience characteristics. For example, if a participant displayed multiple resilience factors (such as social support and problem‐solving) and few negative factors (such as rumination), they would be classified as displaying high resilience. The interview data used to categorize participants were compared to the stress and coping questionnaire data regarding children's resilience, as rated by themselves and their parents, and the groupings were corroborated by the questionnaire data.

#### Coding and screening of data

A life events score was calculated based on a weighting system with higher scores allocated for more serious stressors (Holmes & Rahe, 1967). Daily hassles were summed to obtain a total score. Coping data were split into two scores (frequency and efficacy of coping strategies) for three coping styles (problem‐focussed, emotion‐focussed, and avoidant). This three‐factor model of coping has been identified and tested in previous research using factor analysis (Turner‐Cobb & Steptoe, 1998). Data collected in the demographic questionnaire about parental sex, marital status, occupation, and education were used to create a four‐factor SES score (Hollingshead, 1975).

Data screening identified four participants with outlying salivary cortisol values. The extreme values for three of these participants were recoded to the next highest value (Field, 2009). The fourth participant showed extreme values for multiple cortisol and heart rate values and was therefore excluded from the analysis. Therefore, the final sample size consisted of 33 participants. The cortisol data at the five time points were transformed using a Log10 transformation due to non‐normal distribution (positive skew).

### Statistical analysis

Cortisol was examined across the five time points using paired *t*‐tests. Bonferroni correction was applied to account for multiple comparisons with acceptable significance determined at *p *<* *.005 for cortisol effects and at *p *<* *.017 for analyses with heart rate as the DV. Differences in cortisol levels across the time points were analysed using a MANOVA in which age group, sex, and prior stress–resilience group were entered as the IVs and cortisol at the five time points were entered as the DVs. Relationships between the psychosocial questionnaire data (stress and coping) were analysed using bivariate correlations and ANOVAs.

## Results

Table 2 displays the descriptive statistics for questionnaire data regarding life events, daily hassles, and coping in the four prior stress–resilience groups.

**Table 2:** Means and standard deviations (*SD*) for life events, daily hassles and coping strategies in each of the four prior stress–resilience groups (*n *=* *33)

	High resilience high stress	High resilience low stress	Low resilience high stress	Low resilience low stress
Total severity of life events	218.63 (36.45)	80.10 (37.63)	256.67 (48.15)	62.50 (41.86)
Total number of hassles	45.38 (17.19)	30.80 (16.67)	44.11 (17.88)	40.83 (21.44)
Frequency of coping styles^a^				
Problem‐focussed	3.38 (0.74)	2.10 (1.20)	1.89 (1.17)	2.50 (1.64)
Emotion‐focussed	3.13 (0.84)	3.70 (1.42)	2.78 (1.39)	3.00 (1.10)
Avoidant	2.00 (0.93)	1.70 (0.95)	1.33 (0.87)	1.83 (1.17)
Efficacy of coping styles^b^				
Problem‐focussed	4.75 (1.28)	2.80 (2.10)	2.78 (2.22)	3.83 (2.79)
Emotion‐focussed	2.88 (2.10)	2.70 (1.89)	2.44 (1.24)	3.33 (2.34)
Avoidant	2.75 (1.75)	1.80 (1.68)	1.33 (1.33)	1.50 (1.23)

Notes

a Higher scores represent more frequent usage.

b Higher scores indicate higher perceived usefulness.

### The impact of prior stress–resilience group, sex and age group on salivary cortisol

**Figure 1:**
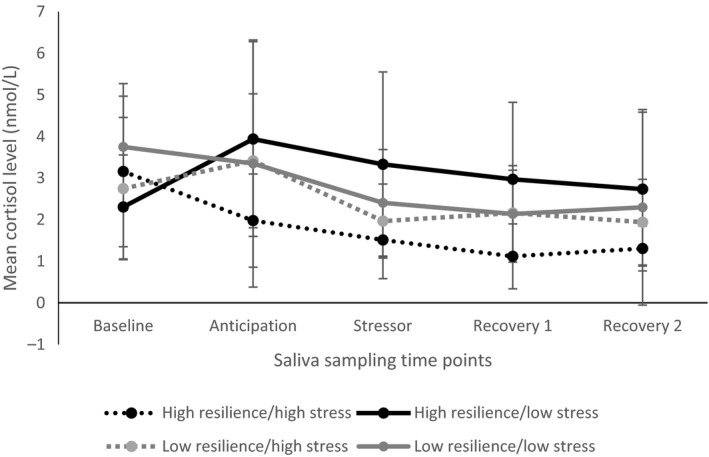
Salivary cortisol levels across the five time points for the four prior stress‐resilience groups (error bars display standard error of the mean).

Salivary cortisol response patterns across the five time points in the four prior stress–resilience groups are illustrated in Figure 1. A series of *t‐*tests revealed salivary cortisol in the anticipation period (*M* = 0.42 nmol/l) to be significantly higher than in the 10 min post‐test (*M* = 0.27 nmol/l), *t*(32) = 3.551, *p *=* *.001, higher in the anticipation period (*M* = 0.42 nmol/l) than 20 min post‐test (*M* = 0.23 nmol/l), *t*(32) = 4.305, *p *<* *.001, and higher in the anticipation period (*M* = 0.42 nmol/l) than 35 min post‐test (*M* = 0.21 nmol/l), *t*(32) = 4.310, *p *<* *.001. There were no significant differences between baseline cortisol levels and other time periods. There were no significant differences between the stressor period (10 min post‐test) and other time periods, other than the anticipation period as detailed above.

MANOVA and follow‐up ANOVAs showed a significant main effect of sex but no significant effects of prior stress–resilience group or age group. Pillai's trace was selected as the test statistic as it is the most robust to violations of assumptions when sample sizes are equal (Field, 2009). Using Pillai's trace, there was a significant effect of sex on salivary cortisol levels, *V *=* *.861, *F*(5, 5) = 6.21, *p *=* *.033, $\eta_{p}^{2}$ = .861. Follow‐up paired samples *t*‐tests showed that males had significantly higher salivary cortisol during the anticipation period (*M* = 0.50 nmol/l) than 20 min post‐test (*M* = 0.25 nmol/l, *p *=* *.002), and higher salivary cortisol in the anticipation period (*M* = 0.50 nmol/l) than 35 min post‐test (*M* = 0.27 nmol/l, *p *<* *.001).

There was a significant interaction between sex and age group for salivary cortisol at baseline, *F*(1, 33) = 8.38, *p *=* *.018, $\eta_{p}^{2}$ = .482; however, follow‐up analyses by sex found no statistically significant differences in salivary cortisol levels between age groups.

There was a significant difference in salivary cortisol level 20 min post‐test between the high resilience/high prior stress experience (*M* = −0.05) and high resilience/low prior stress experience (*M* = 0.40) groups (*p *=* *.044, 95% CI 0.01–0.88). Means show salivary cortisol as highest in the low prior stress group, suggesting that in highly resilient children, experience of stress was associated with lower salivary cortisol in the recovery period.

### The impact of prior stress–resilience group, sex, and age group on heart rate

**Figure 2:**
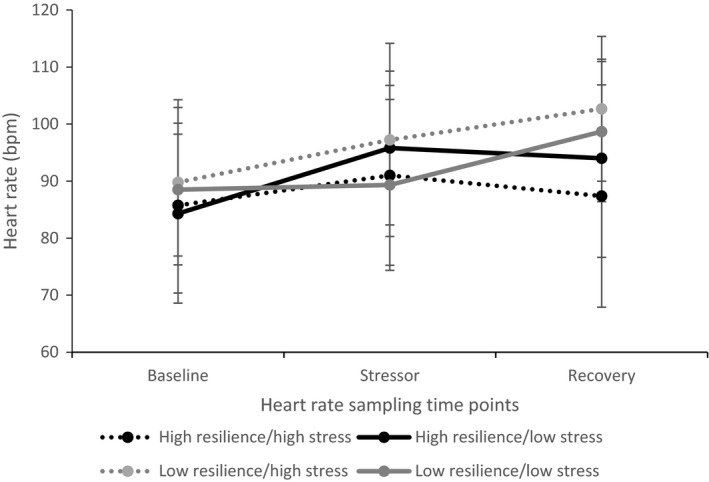
Heart rate values across the three time points for the four prior stress‐resilience groups (error bars display standard error of the mean).

Figure 2 shows the heart rate response patterns across the three time points in the four prior stress–resilience groups. The two high resilience groups show an increase in heart rate from baseline in response to the stressor and decrease during the recovery period (the expected pattern of response). However, the two low resilience groups showed a continued increase in heart rate from the stressor into the recovery period, indicating continued stress arousal. Paired samples *t*‐tests showed a significant difference between heart rate at baseline (*M* = 87 bpm) and during the stressor (*M* = 94 bpm), *t*(32) = −2.818, *p *=* *.008, and heart rate at baseline (*M* = 87 bpm) and during the recovery period (*M* = 96 bpm), *t*(32) = −3.218, *p *=* *.003, but no significant difference between heart rate during the stressor and the recovery period.

A MANOVA showed no significant main effects or interactions for prior stress–resilience group, sex, or age group on heart rate across the three time points. Paired samples *t*‐tests revealed that only nine‐year‐olds showed significantly higher heart rate during the stressor (*M* = 100 bpm) than at baseline (*M* = 88 bpm), *t*(6) = −10.42, *p *<* *.001.

### The post‐stressor verbal subjective report as a manipulation check

In the present study, the BEST‐C did not increase salivary cortisol levels during the stress test as expected, although there was an increase in the anticipation period prior to the task. Therefore, the post‐stressor verbal reports were examined to provide a potential explanation for this finding. Thirty‐one of the 34 participants mentioned feeling scared, worried or nervous during the task, suggesting that the task was stressful, but this stress was not reflected in their salivary cortisol response.

Children made many references to the researcher as a source of social support. For example, one participant reported that they coped with the task by looking at the researcher, two children pretended the researcher was the only person present during the task so it was more like a conversation, and two children said that they did not feel nervous because they were familiar with the researcher. Four participants mentioned that they would have coped less well with the task if there had been more than two people in the panel, suggesting that they did not acknowledge the researcher as the third member of the panel.

### Characteristics of the four prior stress–resilience groups

Problem‐focussed coping was used most frequently in the high resilience/high prior stress group, whereas the other three groups used emotion‐focussed coping most often. All four groups reported that problem‐focussed coping was the most useful coping strategy.

Grouping participants based on high and low levels of resilience highlighted some key differences between the children in these groups. Almost all resilient individuals used a combination of internal and external coping strategies; a conceptualization of coping based on previous research (Radnitz & Tiersky, 2007). Internal coping strategies included personal characteristics and approaches that that did not require input from others, such as positive reframing of situations, patience, and personality features such as a calm and even temperament. External coping strategies included approaches that involved other people, such as talking to parents, siblings, friends and teachers with the view that talking was a way of unburdening oneself and letting go of problems. As detailed in the section above, many of the participants reported finding the researcher to be a source of social support, therefore relying on external coping strategies for managing this particular stressor.

## Discussion

### Physiological and psychological responses to the BEST‐C

Responses to the BEST‐C differed between the four prior stress–resilience groups, with children who had previously experienced more stress and had more characteristics of resilience displaying the lowest salivary cortisol responses to the task. There were no statistically significant increases in salivary cortisol in response to the BEST‐C stress testing phase, contrary to previous findings (Cheetham & Turner‐Cobb, 2016). However, heart rate did increase in response to the task and 31 out of 34 participants self‐reported that they found the task stressful or worrying and were relieved when it was over. These findings suggest that the task was effective at inducing feelings of stress, although this subjective stress did not translate into a salivary cortisol response. Further support for the stressful nature of the task came from the anticipation effect. There was an increase in salivary cortisol in anticipation of the task, suggesting the prospect of completing the BEST‐C was stress‐inducing. This anticipation effect is in line with findings regarding anticipation stress (Cheetham & Turner‐Cobb, 2016). Heightened levels of salivary cortisol during the anticipation period could also explain why salivary cortisol did not increase further during the task as peak salivary cortisol levels may have been reached.

Males showed higher levels of salivary cortisol than females in the post‐test recovery period, suggesting that males continued to feel stressed for longer after the stressful experience, whereas females recovered more quickly. This corroborates previous findings (Cheetham & Turner‐Cobb, 2016) with research suggesting that males display higher heart rate than females when recovering from stress because they use less emotion‐focussed strategies to cope with stressors (Connor‐Smith, Compas, Wadsworth, Thomsen, & Saltzman, 2000). Other research using the TSST‐C in adolescents noted the importance of focussing on age and sex differences in the recovery period to better understand the nuances of children's stress responses (Ji, Negriff, Kim, & Susman, 2015).

### Protective factors in coping with the BEST‐C

The lack of a salivary cortisol stress response to the BEST‐C is likely explained by the presence of the researcher acting as social support due to the relationship and rapport established between participants and the researcher during phase one of the study. A key methodological difference between the present study and the original BEST‐C study was the level of interaction between the participants and the researcher. In the previous study, the participants met the researcher immediately before completing the BEST‐C and so the researcher was a stranger to participants (Cheetham & Turner‐Cobb, 2016). However, in the present study, the participants had met and been interviewed by the researcher prior to completing the BEST‐C. The interviews were mostly conducted in participants’ homes; therefore, they met the researcher in familiar surroundings and developed a rapport during the interview. This combination allowed for the children to feel that the researcher was familiar enough to them to provide comfort or a distraction during the BEST‐C. It was not thought that prior contact with the researcher would have an impact on the outcome of the stress test; however, in the interviews, when children were asked how they coped with the task, many of them referred to the presence of the researcher.

Social support has been examined directly elsewhere in the stress testing literature. For example, research with adults has compared the regular TSST paradigm with a friendly version (the f‐TSST) in which the audience were friendly and encouraging towards the participant during the task (Wiemers, Schoofs, & Wolf, 2013). The TSST induced social evaluative threat and increased cortisol, whereas the f‐TSST did not increase cortisol in adults, suggesting that acting positively towards participants can be an effective stress buffer. Although the researcher in the present study acted in a neutral manner during the task, their previous rapport with the participants during the interviews may have been focused on by the participants during the BEST‐C. This unintended finding demonstrates a very important phenomenon and may help to explain other ambiguous findings in the stress testing literature, as well as offering potential avenues for stress reduction interventions.

### Acute stress responses differ based on prior stress experience and resilience

Coding the parent–child dyadic interviews conducted with this population in phase one of the study was an effective way to establish the impact of stress experience and resilience characteristics on children's acute stress responses. Significant differences were highlighted in the salivary cortisol data across the four prior stress–resilience groups, all of whom experienced an acute social stressor in the context of social support. In the two groups of highly resilient children, those who had experienced high levels of stress displayed the lowest levels of salivary cortisol across the time points. This suggests that when social support is available during an acute stressor, a resilient character and prior experience of managing stress means children are better able to cope. The highly resilient children who had experienced lower levels of stress in the past year showed the highest levels of salivary cortisol throughout the task, further supporting the proposal that experience of stress can be beneficial when it comes to dealing with a social stressor. The two low resilience groups showed similar patterns across the time points indicating that, in terms of their salivary cortisol responses to an acute stressor, experience of stress was not a distinguishing factor for individuals with low levels of resilience.

Previous research has also focussed on how resilience can impact acute stress responses, for example characteristics of resilience such as personality and temperament have been found to impact how children respond to the TSST (Childs, White, & de Wit, 2014; Tyrka *et al*., 2007). Similarly, research has considered how temperament can enhance stress–resilience (Smith & Prior, 1995; Yendork & Somhlaba, 2015) and how distinguishing between resilient and stress‐affected adolescents helps us to better understand the protective resources which can be used to moderate stress responses (D'Imperio *et al*., 2000).

### Strengths and limitations

A mixed methods analytic approach using quantitative coding of parent–child dyadic interviews enabled a fuller assessment of children's acute stress responses contextualized by their experiences of stress and the resilience characteristics they possessed. The coding was theoretically sound as it was based on a framework used successfully with adolescents (D'Imperio *et al*., 2000). This study built on previous research by using the BEST‐C protocol, and addressed issues raised in the initial research, such as collecting a baseline saliva sample on a control day before the experimental procedures. The findings from this study indicate that social support and resilience characteristics are protective factors in stress management. Interventions, such as toolkits, designed to help children manage stress should acknowledge these individual differences.

However, the study does have its limitations. The main finding from previous research, that the BEST‐C induced a salivary cortisol response in children (Cheetham & Turner‐Cobb, 2016), was not supported. This can be explained in relation to emotional social support and the impact this has on children's acute stress response; an important finding in its own right. Although an unintended outcome, the researcher acting as a form of social support advanced the research by demonstrating the effectiveness of social support as a buffer to social stress.

A limitation and important consideration for future research is the methodological issue surrounding heart rate measurement in children. Heart rate was measured for half an hour and every effort was made to ensure that conditions were consistent throughout the period of measurement. However, due to the nature of laboratory stress testing, children were seated while their heart rate was measured during the baseline and recovery periods, whereas they were stood up in front of the audience during the BEST‐C (as this is part of standard stress testing protocol). Participants also walked between the waiting room and the stress lab before the BEST‐C and to the debrief room after the task. Both standing up and walking around could have elevated children's heart rate therefore the heart rate findings must be treated with caution (Strahler, Mueller, Rosenloecher, Kirschbaum, & Rohleder, 2010). Future research could ensure more consistency in heart rate measurements by having participants sit during the 30 min of measurement, so that the anticipation, stressor, and recovery period all take place in the same location to avoid the participant walking while wearing the heart rate monitor.

In sum, the present study highlights the relevance of contextual factors such as prior stress experience, characteristics of resilience, and exposure to social support in understanding children's physiological responses to the anticipation of an acute social stressor. The context of social support provides a valuable lens through which to view social stress testing. Findings provided support for the capacity of the BEST‐C protocol to induce an anticipation stress response in children aged 7–11 years. This two‐phase method of data collection and mixed methods analysis allowed for an in‐depth investigation of children's wider experiences of stress, resilience characteristics and how these factors related to their responses to an acute stressor.

## Conflict of interest

All authors declare no conflict of interest.
